# The Neglected Art of Prescription Writing

**Published:** 1907-11-02

**Authors:** 


### The Neglected Art of Prescription Writing.

The medical profession owes gratitude to those who point out faults and failings in the curriculum, suggesting, at the same time, remedies. Destructive criticism is the easiest of arts, and the pitfall of the unwise is a glib facility in attack. No profession is exempt from the criticaster, no curriculum secure against the assault of the would-be reformer; and the profession of medicine, because it lends itself especially to criticism from within outwards, stands in the unhappy position of being one of the largest giants for the reforming Don Quixote to break lance against. Layman and leech have had their say against the shortcomings of the medical curriculum, some unwisely, others with a proper regard for the fitness of things, looking at the whole from a true perspective, and neither through a veil nor through the rose-coloured glasses of interested prejudice. Criticism that comes from the latter class is always welcome, for it is usually instructive, and expressed in a kindly, generous way that wins support from the start because it disarms opposition by very kindliness. We confess, therefore, to a feeling of cordial gratitude to Dr. James Burnet, whose thoughtful little pamphlet on "The Passing of the Prescription: Its Causes and Effects," deserves the meditative attention of every medical man, and, let us add, of every medical student. The subject with which Dr. Burnet deals is one that cannot fail to interest professional readers. It is the decay in the art of prescription writing—the growing tendency on the part of the practitioner to take refuge in the shallows of the manufacturing chemist's catalogue—the complaint that the apothecary has started to prescribe because the medical practitioner has ceased to do so. With Dr. Burnet's premises every thoughtful man will agree. He finds the cause for this lamentable decline in the study of prescription writing in the neglect of adequate instruction in the essentials by teachers of pharmacy and therapeutics, in the use of stock preparations at hospitals and infirmaries, and in the aggressive advertising of the manufacturing chemist, to which sooner or later most medical practitioners fall victims. "Many of our younger men," he remarks, "are so intent nowadays upon serums and vaccines, or opsonins and blood counts, that they are apt to run after shadows while they neglect the substance." The indictment is a true one, and still more true is the added item that many teachers are utterly incapable of teaching their pupils to write out prescriptions correctly because they themselves have never been properly trained in the art. The cynic might retort to all this that a knowledge of pharmaceutical Latin has never been the glory of the profession, and that even in the days when poly-pharmacy was the hall-mark of success in a specialist, some of the leading lights in the profession wrote prescriptions such as "no second year's student in *materia medica* would be allowed to write."

But the evil that Dr. Burnet exposes so ably does not, indeed, consist only, or merely, of an inability to write the correct Latin equivalent for gelatine, linseed poultice, or castor oil: it goes deeper, for its tendency is to undermine the relations that should exist between the practitioner and his patient. Once the former has thrust the latter into the hands of the chemist by "prescribing" somebody's tabloids he loses, to a large extent, his hold upon the patient. There is much to be said in favour of the theory that the patient should not be told what he is taking: there is little, if anything, to support the opinion that the doctor should allow the chemist to treat the patient, or the patient's friends, upon a prescription given for a particular condition or complaint. The whole question of prescription writing, and the making up of medicines, needs to be carefully and dispassionately discussed by the profession, and we welcome Dr. Burnet's suggestion that a medical society should take up the matter and thresh it out in conjunction with the chemist. The remedy lies less, we think, in inculcating into the student a proper knowledge of dog-Latin than in making him take an active interest in drug treatment from the very start. It certainly does not lie in teaching the coming practitioner to despise serums, opsonins and compressed drugs, so long as he is taught that these things, like all others, have their limitations, drawbacks, and disadvantages. Rather does it lie in educating the profession to realise the extent of the evil that is already upon us, and to impress upon each student, before yet he leaves the schools, the fact that his duty to the patient does not cease the moment he has handed the case over to the chemist.

